# Preoperative Mechanical Ventilation Prior to Surgical Repair for Type A Aortic Dissection: Incidence, Risk, and Outcomes

**DOI:** 10.3390/jcdd12070239

**Published:** 2025-06-23

**Authors:** Angelo M. Dell’Aquila, Konrad Wisniewski, Adrian-Iustin Georgevici, Gábor Szabó, Francesco Onorati, Till J. Demal, Andreas Rukosujew, Sven Peterss, Caroline Radner, Joscha Buech, Antonio Fiore, Andrea Perrotti, Angel G. Pinto, Javier Rodriguez Lega, Marek Pol, Petr Kacer, Enzo Mazzaro, Giuseppe Gatti, Igor Vendramin, Daniela Piani, Luisa Ferrante, Mauro Rinaldi, Eduard Quintana, Robert Pruna-Guillen, Dario Di Perna, Zein El-Dean, Hiwa Sherzad, Giovanni Mariscalco, Mark Field, Amer Harky, Manoj Kuduvalli, Matteo Pettinari, Stefano Rosato, Tatu Juvonen, Timo Mäkikallio, Lenard Conradi, Giorgio Mastroiacovo, Fausto Biancari

**Affiliations:** 1 Department of Cardiac Surgery, University Hospital Halle, 06120 Halle, Germany; gabor.szabo@uk-halle.de; 2 Department of Cardiothoracic Surgery, University Hospital Muenster, 48149 Muenster, Germany; konrad.wisniewski@ukmuenster.de (K.W.); andreas.rukosujew@ukmuenster.de (A.R.); 3 St. Josef-Hospital, University Hospital of Ruhr-University of Bochum, 44791 Bochum, Germany; adrian.georgevici@uk-halle.de; 4 Division of Cardiac Surgery, University of Verona Medical School, 37129 Verona, Italy; francesco.onorati@univr.it; 5 Department of Cardiovascular Surgery, University Heart & Vascular Center Hamburg, 20251 Hamburg, Germany; t.demal@uke.de; 6 Department of Cardiac Surgery, LMU University Hospital, Ludwig Maximilian University, 81337 Munich, Germany; sven.peterss@med.uni-muenchen.de (S.P.); caroline.radner@med.uni-muenchen.de (C.R.); joscha.buech@med.uni-muenchen.de (J.B.); 7 German Centre for Cardiovascular Research, Partner Site Munich Heart Alliance, 80802 Munich, Germany; 8 Department of Cardiac Surgery, Hôpitaux Universitaires Henri Mondor, Assistance Publique-Hôpitaux de Paris, 94000 Creteil, France; fioreant7@yahoo.com; 9 Department of Thoracic and Cardiovascular Surgery, University of Franche-Comte, 25030 Besancon, France; a.perrotti@hotmail.it; 10 Cardiovascular Surgery Department, University Hospital Gregorio Marañón, 28007 Madrid, Spain; angel.g.pinto@gmail.com (A.G.P.); jrl2003@gmail.com (J.R.L.); 11 Department of Cardiac Surgery, Third Faculty of Medicine, Charles University and University Hospital Kralovske Vinohrady, 10000 Prague, Czech Republic; pol.marek@email.cz (M.P.); petr.kacer@fnkv.cz (P.K.); 12 Division of Cardiac Surgery, Cardio-thoracic and Vascular Department, Azienda Sanitaria Universitaria Giuliano Isontina, 34149 Trieste, Italy; enzo.mazzaro@asugi.sanita.fvg.it (E.M.); gius.gatti@gmail.com (G.G.); 13 Cardiothoracic Department, University Hospital of Udine, 33100 Udine, Italy; igor.vendramin@asufc.sanita.fvg.it (I.V.); danielapiani89@gmail.com (D.P.); 14 Cardiac Surgery, Molinette Hospital, University of Turin, 10126 Turin, Italy; luisa.ferrante@unito.it (L.F.); mauro.rinaldi@unito.it (M.R.); 15 Department of Cardiovascular Surgery, Hospital Clínic de Barcelona, University of Barcelona, 08036 Barcelona, Spain; equintan@clinic.cat (E.Q.); pruna@clinic.cat (R.P.-G.); 16 Department of Cardiac Surgery, Centre Hospitalier Annecy Genevois, 74370 Epagny Metz-Tessy, France; 0dariodiperna0@gmail.com; 17 Department of Cardiac Surgery, Glenfield Hospital, Leicester LE3 9QP, UK; zein.eldean@uhl-tr.nhs.uk (Z.E.-D.); hiwa.sherzad@uhl-tr.nhs.uk (H.S.); giovannimariscalco@yahoo.it (G.M.); 18 Liverpool Centre for Cardiovascular Sciences, Liverpool Heart and Chest Hospital, Liverpool L14 3PE, UK; mark.field@lhch.nhs.uk (M.F.); ameralihasan.harky@lhch.nhs.uk (A.H.); manoj.kuduvalli@lhch.nhs.uk (M.K.); 19 Department of Cardiac Surgery, Ziekenhuis Oost Limburg, 3600 Genk, Belgium; 20 National Center for Global Health, Istituto Superiore di Sanitá, 00161 Rome, Italy; stefano.rosato@iss.it; 21 Research Unit of Surgery, Anesthesia and Critical Care, University of Oulu, 90570 Oulu, Finland; tatu.juvonen@hus.fi; 22 Heart and Lung Center, Helsinki University Hospital, University of Helsinki, 00290 Helsinki, Finland; 23 Department of Medicine, South-Karelia Central Hospital, University of Helsinki, 53130 Lappeenranta, Finland; timo.makikallio@sydankeskus.com; 24 Department of Cardiac Surgery, Cologne University Hospital, 50937 Cologne, Germany; lenard.conradi@uk-koeln.de; 25 Department of Cardiovascular Surgery, Centro Cardioologico Monzino IRCCS, 20138 Milan, Italy; giorgio.mastroiacovo@cardiologicomonzino.it (G.M.); fausto.biancari@cardiologicomonzino.it (F.B.)

**Keywords:** type a aortic dissection, invasive mechanical ventilation, intubation

## Abstract

Objectives: Several conditions associated with type A aortic dissection may require preoperative invasive mechanical ventilation (IMV). The current literature lacks data on this subset of patients’ prevalence and postoperative outcomes. This study aims to investigate this unexplored issue in a multicenter European registry. Methods: Data from 3735 patients included in the European Registry of Type A Aortic Dissection (ERTAAD) were the subject of this analysis. Bootstrapped Least Absolute Shrinkage and Selection Operator (LASSO) logistic regression was performed for variable selection to identify key predictors of hospital death. In the second step, a multilevel multivariable logistic regression (MMLR) was carried out, given the clustered structure of the data. Results: A total of 346 (9.3%) out of 3735 patients required preoperative IMV. Compared to the non-IMV patients, patients requiring IMV had a significantly higher rate of organ malperfusion (52% vs. 35%, *p* < 0.001) and a higher proportion of tears in the aortic root (*p* = 0.048). The in-hospital mortality rate among IMV patients was 38% vs. 15% in non-IMV patients (*p* < 0.001), without a difference in post-discharge survival (*p* = 0.84). At the MMLR, patients who required IMV had 135% higher odds of in-hospital death compared to the remaining patients. IMV yielded the second highest odds in the prediction model for in-hospital mortality (OR 2.13, CI 1.60 to 2.85, *p* < 0.001). Among IMV patients, the extension of surgery to the aortic arch was significantly associated with increased in-hospital mortality (*p* < 0.001, OR 2.98). In multivariable analysis, preoperative IMV was independently associated with increased odds of in-hospital mortality. Conclusions: The need for invasive mechanical ventilation before surgical repair for type A aortic dissection is not infrequent. In this subpopulation, the in-hospital mortality rate was twofold compared to patients who did not require IMV. The awareness of the preoperative risk profile and outcomes of this subset of patients should urge surgeons to tailor the surgical strategy more appropriately to improve the immediate postoperative results.

## 1. Introduction

Surgical repair of acute type A aortic dissection is still associated with significant mortality rates. In this regard, large registries report early mortality rates of about 18% [1,2,3]. Many conditions such as hemodynamic instability and malperfusion syndrome may require preoperative invasive mechanical ventilation (IMV). In this setting, only two single-center studies validating the GERAADA score reported general data on this subset of patients, including prevalence and mortality rates [4,5]; however, there is a lack of specific clinical data on baseline characteristics, complications, and late follow-up of this subgroup of patients.

Thus, the current study aims to investigate this unexplored issue in a large European multicenter registry.

## 2. Patients and Methods

### 2.1. Study Population

The present analysis is based on the data of 3735 consecutive patients from the European registry of type A aortic dissection (ERTAAD). This comprises data from a multicenter, retrospective cohort including consecutive patients who received an operation for acute TAAD at 17 centers of cardiac surgery located in eight European countries (Belgium, Czech Republic, Finland, France, Germany, Italy, Spain, and the United Kingdom) from January 2005 to March 2021. One center was excluded due to non-compliance with the internal protocol regarding data collection and lack of approval for submission.

The Institutional Review Board and Ethics Committee of the University Hospital Muenster (18 June 2021, diary no. 2021-368-f-S) approved this retrospective study. According to the approval, individual informed consent was not required in this retrospective analysis and therefore waived. The ERTAAD was registered in ClinicalTrials.gov with the identifier NCT04831073 (https://clinicaltrials.gov/study/NCT04831073; accessed on 29 March 2021).

### 2.2. Definition of Preoperative Invasive Mechanical Ventilation

In this study, preoperative invasive mechanical ventilation (IMV) was defined as mechanical ventilation via orotracheal intubation initiated prior to arrival in the operating room for surgical repair. Patients were intubated in the pre-hospital setting, emergency department, or intensive care unit, based on clinical indications such as respiratory failure, hemodynamic instability, neurological compromise, or peri-arrest conditions. No patients classified as preoperative IMV were extubated before surgery; all remained intubated until surgical intervention. Data on the precise duration of preoperative IMV were not available due to registry limitations.

### 2.3. Data Preprocessing

Continuous variables are presented as medians and interquartile differences. Categorical variables are shown as counts and percentages. Differences between IMV and non-IMV patients were obtained using the Wilcoxon rank sum test for continuous variables, whereas the Chi-square test was applied for categorical variables when all expected cell counts were ≥5, and Fisher’s test was used for categorical variables when any expected cell count was <5.

For the correct fitting of the multilevel multivariable linear model with grouping by center, we assessed missing data for each variable across centers. Variables with less than 75% non-missing data in any center were excluded to ensure sufficient information within each group. Additionally, to maintain the model’s validity, we excluded variables with insufficient variance in at least one center. Specifically, variables that showed zero variance per outcome in any center were removed. This approach ensured that each variable included in the model had adequate variability across all centers, supporting the robustness of the multilevel analysis. Redundant variables with a correlation coefficient close to 1 were also removed to prevent multicollinearity issues in the subsequent linear models. Missing values in numeric variables were imputed using the median of the respective variable within each center. Each non-ordinal categorical variable was decomposed into multiple binary variables for each level. The ‘Year’ variable was rescaled to the range of 0 to 1; all other continuous variables were standardized to have a mean of 0 and a standard deviation of 1, thereby enhancing the model’s interpretability. Furthermore, we performed a bootstrapped Least Absolute Shrinkage and Selection Operator (LASSO) logistic regression for variable (feature) selection to identify key predictors of hospital death and remove non-predictive variables. LASSO works by applying a penalty lambda to the size of the coefficients of the variables, shrinking less important ones to zero [6]. The bootstrapping used stratified sampling by both center and in-hospital death outcomes to ensure the preservation of covariate distributions. A LASSO grid search for optimal lambda was performed to maximize the receiver operating characteristic (ROC) area under the curve (AUC). Variables with coefficients corresponding to odds ratios between 0.9 and 1.1 were excluded, retaining only those significantly associated with hospital death. After applying the variable exclusion criteria based on insufficient variance and missing values described in Section 2, the following variables were retained for analysis: hospital death, center, onset of symptoms to surgery hours, year, age, surgery during night-time (20:00–08:00), female, eGFR CKD-EPI, arterial lactate, hypertension, pulmonary disease, cardiogenic shock requiring inotropes, Penn classification, urgency of the procedure, any malperfusion, cerebral malperfusion, peripheral malperfusion, aortic valve insufficiency, common femoral artery cannulation, tear in aortic root, tear in ascending aorta, dissection of aortic root involving non-coronary cusp, number of segments of aortic root involved, supracoronary replacement, aortic root replacement, Bentall procedure, aortic valve replacement type, type of aortic arch repair, total or partial aortic arch repair, total aortic arch repair, myocardial ischemic time, total aortic arch replacement, deep to moderate hypothermic circulatory arrest, lowest temperature during hypothermic circulatory arrest, antegrade cerebral perfusion time, antegrade cerebral perfusion, left common carotid artery antegrade, and invasive mechanical ventilation.

The final step of our analysis consisted of the multilevel multivariable logistic regression (MMLR), which is advantageous for clustered data, such as patients nested within centers. This modeling approach allows for the inclusion of random effects, such as the random intercept for the center in this study, which accounts for variability between centers and controls for potential center-specific differences that might influence outcomes. By considering both the individual- and group-level variations, multilevel models provide more accurate and generalizable estimates of predictor effects, provided that non-relevant and highly collinear variables are dropped [7]. Hence, only the LASSO-selected variables in the precedent step were included for the fitting of MMLR.

A sub-analysis including only IMV patients was conducted using a logistic regression model to identify risk factors for in-hospital mortality.

Survival estimates were generated for the long-term cohort using Kaplan–Meier analysis. Cox regression analysis, including only discharged patients, was performed to explore differences in late survival between IMV and non-IMV patients after discharge. The statistical analyses were performed using the R programming language [8] with the tidyverse package for data preprocessing [9], glmnet packages for the tuned LASSO regression [10], and the lme4 package for fitting multilevel logistic regression models [11].

In summary, variable selection for multivariable modeling was performed using bootstrapped Least Absolute Shrinkage and Selection Operator (LASSO) logistic regression, with stratified sampling by center and in-hospital outcomes. This approach allowed us to identify the most relevant predictors and reduce the risk of overfitting or collinearity. Subsequently, a multilevel multivariable logistic regression (MMLR) model was constructed to account for the hierarchical, multicenter data structure and adjust for potential center-level effects. Variance inflation factors (VIFs) were calculated to confirm the absence of problematic multicollinearity among the included variables (see Supplementary Material).

## 3. Results

### 3.1. Patients’ Characteristics

A total of 346 (9.3%) out of 3735 patients required preoperative IMV. A detailed flowchart depicting inclusion and exclusion criteria and the final study population is provided in the Supplementary Material. Compared to the non-IMV patients, patients requiring IMV were older (67 vs. 64 years, *p* = 0.02) and had a significantly worse renal function (eGFR—CKD-EPI, 70 vs. 66 mL/min, *p* = 0.038) and higher preoperative arterial lactate values (*p* < 0.001). Patients requiring IMV had significantly higher rates of preoperative cardiac massage (20% vs. 3%, *p* < 0.001) and cardiogenic shock requiring inotropes (50% vs. 14%, *p* < 0.001). IMV patients had a significantly higher rate of organ malperfusion (52% vs. 35%, *p* < 0.001). Specifically, the rate of cerebral malperfusion in IMV patients was almost double that in non-IMV patients (39% vs. 20%, *p* < 0.001). Table 1 shows the differences at baseline between the study groups. IMV patients had a significantly higher proportion of tears in the aortic root (*p* = 0.048). However, root replacement was performed in those patients less frequently (25% vs. 29%, *p* = 0.082). Table 2 shows the intraoperative differences between the study groups.

### 3.2. Postoperative Complications and Prediction of Mortality

IMV patients experienced significantly higher rates of global brain ischemia and hemorrhagic stroke (*p* < 0.001), whilst the rate of postoperative ischemic stroke was comparable (*p* = 0.8) between the study groups. Mesenteric ischemia and temporary dialysis were significantly higher in IMV patients (*p* = 0.01 and *p* < 0.001, respectively). Moreover, IMV patients had about a 4-fold higher incidence of venoarterial ECMO postoperatively (8.1% vs. 2.4%, *p* < 0.001) compared to non-IMV patients.

The mortality rate among IMV patients was 38% vs. 15% in non-IMV patients (*p* < 0.001). Data on postoperative outcomes are displayed in Table 3. Baseline differences between survivors and in-hospital deaths are provided in Table 4.

The bootstrapped LASSO, which maintains the original center–outcome ratio, with a lambda penalty of 0.00625, showed an ROC AUC of 0.761 ± 0.014. Patients who received invasive mechanical ventilation had 135% higher odds of in-hospital death compared to patients who were not intubated (*p* < 0.001, OR = 2.13, CI 1.60 to 2.85). In the prediction model, the presence of IMV yielded the highest odds for in-hospital mortality. The following further LASSO variables were identified as risk factors for in-hospital mortality: peripheral malperfusion (OR 1.40, CI 1.09 to 1.80), coronary artery bypass grafting (OR 2.54, CI 1.93 to 3.35), and urgency of the procedure (OR 1.77, CI 1.56 to 2.00) (validated definitions for the urgency of the procedure are given in Table 5 according to the study protocol [12,13]). The latter odds ratio indicates that for each one-level increase in the urgency of the procedure, the odds of in-hospital death increase by 63%. In contrast, the year of operation (OR 0.52, CI 0.37 to 0.74; for each year, the in-hospital mortality decreased by ≈3.9%) and eGFR CKD-EPI (OR 0.63, CI 0.57 to 0.69) were found to be protective. Table 6 shows the results of the LASSO and the MMLR models in detail.

**Figure 1:**
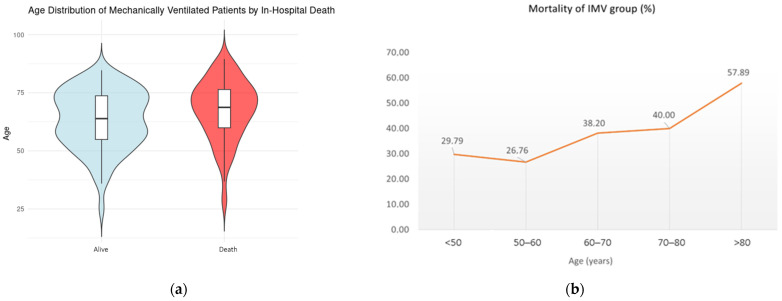
(**a**) Age distribution between survivors and non-survivors (violin diagram); (**b**) trend of mortality according to the increasing age among IMV patients.

Among IMV patients, age (*p* = 0.028, OR 1.02, CI 1.00 to 1.05), the urgency of the procedure (*p* = 0.01, OR 1.65, CI 1.13 to 2.43), the intraoperative need for CABG (*p* = 0.007, OR 3.05, CI 1.37 to 6.95), and the extension of the surgery to the aortic arch (*p* < 0.001, OR 2.98, CI 1.60 to 5.62) were found to significantly increase in-hospital mortality, whereas eGFR CKD-EPI (*p* < 0.001, OR 0.93, CI 0.97 to 0.99) had a protective effect. Figure 1 shows the age distribution between survivors (A) and non-survivors and the trend of mortality according to increasing age among IMV patients (B).

**Figure 2:**
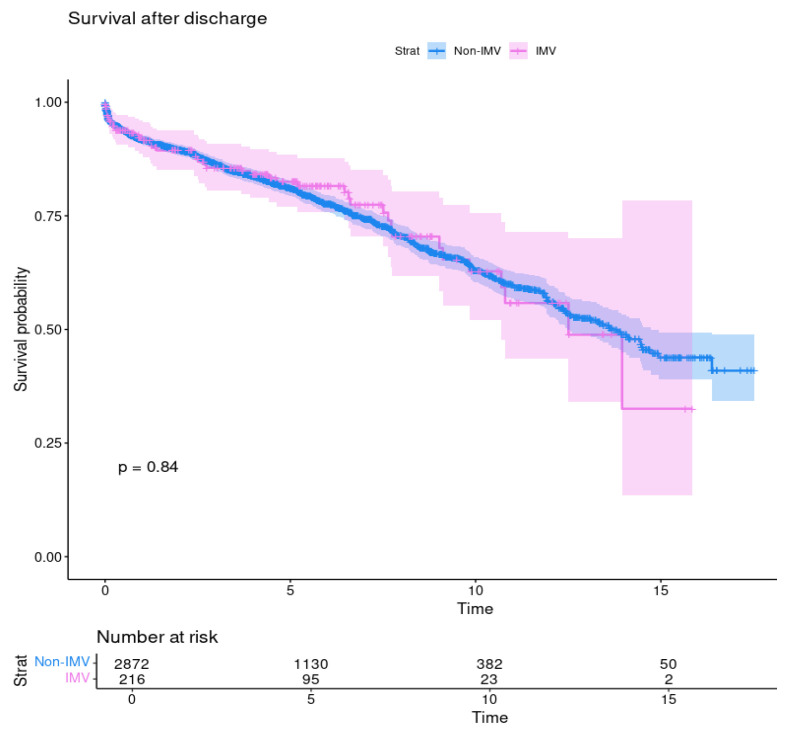
Survival after discharge of patients with preoperative invasive mechanical ventilation (IMV) and without (no-IMV); *p* = 0.84.

After discharge, IMV patients’ survival estimates were 92.2%, 82.5%, and 62.8% vs. 92.9%, 91.7%, and 63.1% from non-IMV patients at 1, 5, and 10 years, respectively. These rates did not differ between the study groups (*p* = 0.84) (Figure 2).

Sensitivity analysis revealed variability in preoperative IMV utilization rates among different centers. Multilevel logistic regression was employed to control for these center-level variations.

## 4. Discussion

Patients who received preoperative IMV had 135% higher odds of in-hospital death compared to patients who did not need to be intubated. This was the highest odds reported in the prediction model for in-hospital mortality. Given this result, it is of utmost importance to consider this condition before patient referral. Hence, IMV is associated with a higher probability of root dissection and a higher rate of malperfusion, which surgeons should consider for proper tailoring of the surgical strategy. In this regard, the first difference between both groups was that there was a significantly lower rate of David procedures in the IMV group (0.6% vs. 4.3%) and the same proportion of Bentall procedures (23% in both groups) despite a significantly higher rate of tears in the aortic root (21% vs. 16%). This supports the thesis that surgeons were less aggressive in sicker patients opting for a less demanding procedure.

Another interesting finding of this study is the significantly higher mortality rate of IMV patients who received an extension of surgery to the arch (25% vs. 17%, *p* < 0.001, OR 2.98). This important finding should lead surgeons to be more conservative in performing distal procedures in view of the threefold increased odds of in-hospital mortality.

Regarding the incidence of IMV and the mortality rate among those patients, in the single-center study of Berezowski including 689 consecutive patients, the incidence was 6.4% and the mortality rate was 34.1%. In the study of Luehr et al. including 371 consecutive patients, the incidence of IMV was 15.1% and the mortality rate was similar to that in our cohort (37.1%). In the present study, we reported an incidence of IMV of 9.3% with a mortality of 38%. Although our mortality rates are in line with the two aforementioned studies, discrepancies regarding the incidence of IMV are likely due to many factors related to the inherent limitations of single-center observations. Those factors may include the lower number of patients and the differences in acceptance and referral of this high-risk subpopulation. Thus, given those limitations, our multicenter report offers a better overview of this subpopulation.

Considering other multicenter reports such as the GERAADA registry, in which a total of 2.137 patients were included, the need for preoperative invasive mechanical ventilation was not retained in the multivariable model for in-hospital mortality (*p* = 0.112) [2]. However, a later publication from the same registry with a higher number of patients (n = 2.537) integrated this variable within the GERAADA score [14]. In the report of Pollari et al. [15], in which four different scores were employed for the prediction of in-hospital mortality, the GERAADA score yielded better performance in the prediction of in-hospital mortality than the IRAD score [16]. Interestingly, the latter does not consider the preoperative need for mechanical ventilation in its calculation. Without speculating that the absence of this variable could have lowered the prediction performance of the IRAD score, we are confident that due to its important contribution to postoperative mortality, IMV should be considered in the development of future risk scoring method scores.

Interestingly, the odds ratio for the 30-day mortality in the GERAADA score was 1.95, and this was similar to our risk estimate (OR 2.20). In this regard, the current study addressed the impact on mortality through very robust statistical methods: the LASSO and the MMLR. The OR differences between the models were relatively small because the LASSO was fitted on bootstrapped data samples that maintained the same mortality ratio per center as in the non-sampled data. This approach ensured that the center-specific differences in associations between covariates and in-hospital mortality were preserved in the LASSO model, contributing to its similar baseline and effect estimates despite not explicitly modeling the hierarchical structure like the MMLR. The slightly higher intercept in the MMLR reflects the model’s adjustment for center-level clustering, which tends to moderate extreme values and capture variations across different centers. In contrast, the LASSO model does not account for this clustering, leading to a slightly lower baseline OR. These differences reflect the role of including random effects in the MMLR, which models the underlying variability between centers, while LASSO provides a more generalized estimation across the entire dataset.

Differences in the observed odds ratios for IMV between this and our previous publications can be explained by variations in statistical methodology, population inclusion criteria, and extent of risk adjustment. The present study utilized a larger multicenter cohort, bootstrapped LASSO variable selection, and multilevel modeling with center-level adjustment, which may have contributed to differences in effect estimates.

Importantly, our findings should not be interpreted as evidence that preoperative invasive mechanical ventilation (IMV) is a direct causal factor for adverse outcomes. Rather, IMV predominantly serves as a marker of increased clinical severity, reflecting profound hemodynamic instability, advanced malperfusion, or neurological compromise. This association is well-recognized in acute cardiovascular emergencies, and our results reinforce the importance of recognizing IMV as an epiphenomenon of underlying disease severity, not an independent risk modifier. Future prospective studies are needed to further elucidate the causal pathways and potential modifiable factors within this high-risk subgroup.

Furthermore, while our multicenter, adjusted analysis provides robust risk stratification, we acknowledge the potential for residual confounding inherent to observational registry studies. The associations identified should therefore be interpreted in the context of these methodological limitations.

To account for between-center variability in IMV utilization and care processes, we employed multilevel logistic regression with random intercepts for center and conducted sensitivity analyses examining center-level IMV rates. Despite these adjustments, residual selection bias may remain and should be considered when interpreting the results.

Although the association between critical illness and worse outcomes is well-recognized, our study is, to our knowledge, the first to provide multicenter, risk-adjusted quantification of mortality risk associated with preoperative IMV in acute type A aortic dissection. IMV emerged as one of the strongest independent predictors of in-hospital mortality in robust multivariable modeling. This quantitative risk stratification may support preoperative counseling, surgical planning, and the refinement of future prognostic scores for TAAD.

These findings suggest that awareness of the significantly increased mortality risk associated with preoperative IMV could assist surgeons in better tailoring surgical strategies, potentially opting for less complex and more conservative operative procedures when clinically feasible.

Given the richness of the ERTAAD dataset, future analyses should be planned to further investigate specific indications for IMV, the duration of preoperative ventilation, and time-dependent effects on outcomes, provided the necessary data granularity is available.

In conclusion, the need for IMV before surgery is the first risk factor for in-hospital mortality, and predictive scores should consider this baseline variable. Moreover, surgeons should consider this preoperative condition for proper tailoring of the surgical strategy to reduce the risk of early postoperative mortality.

## 5. Limitations

This study is limited by its retrospective observational design. Although we performed rigorous multivariable adjustment and utilized advanced statistical modeling to address confounding, residual confounding from unmeasured or incompletely characterized variables may still exist. In particular, the indication and precise timing of IMV initiation, as well as the severity of underlying comorbidities, were not available in all cases and thus could not be fully explored.

Additionally, the classification of patients as “preoperative IMV” may be subject to misclassification bias due to variations in documentation and practice patterns across centers.

Although preoperative pulmonary disease was uniformly defined as the use of bronchodilators and/or steroids according to the study protocol and equally distributed between both groups, the severity of pulmonary disease was not specified or captured, and therefore could not be included in the statistical analysis. Finally, data regarding the precise timing and duration of invasive mechanical ventilation (IMV) prior to surgery were not collected, which precluded a detailed analysis of its impact on outcomes and its inclusion in the regression model.

## Figures and Tables

**Table 1:** Baseline characteristics of the study cohorts.

Baseline Characteristics	Overall *N* = 3735	Non-IMV Patients *N* = 3389	IMV Patients *N* = 346	*p*-Value ^1^
Estimated distance to hospital (km)	28 (8, 60)	27 (8, 60)	30 (10, 65)	0.2
Age (years	64 (55, 74)	64 (54, 74)	67 (56, 75)	0.020
Octogenarians	304 (8.1%)	266 (7.8%)	38 (11%)	0.042
Female	1140 (31%)	1031 (30%)	109 (32%)	0.7
Weight	80 (70, 90)	80 (70, 90)	80 (70, 90)	0.2
Height	174 (165, 180)	174 (165, 180)	174 (166, 180)	0.5
eGFR-CKD-EPI (mL/min/1.73 m^2^)	69 (53, 87)	70 (53, 87)	66 (48, 86)	0.038
Arterial lactate	1.60 (1.00, 2.80)	1.60 (1.00, 2.60)	2.15 (1.20, 4.20)	<0.001
Genetic syndromes	79 (2.1%)	72 (2.1%)	7 (2.0%)	>0.9
Prior cardiac surgery	118 (3.2%)	113 (3.3%)	5 (1.4%)	0.056
Iatrogenic TAAD	102 (2.7%)	89 (2.6%)	13 (3.8%)	0.2
Aspirin	728 (19%)	642 (19%)	86 (25%)	0.008
Oral anticoagulant	248 (6.6%)	229 (6.8%)	19 (5.5%)	0.4
Hypertension	2653 (71%)	2407 (71%)	246 (71%)	>0.9
Diabetes	181 (4.8%)	166 (4.9%)	15 (4.3%)	0.6
Prior stroke	145 (3.9%)	127 (3.7%)	18 (5.2%)	0.2
Pulmonary disease	319 (8.5%)	289 (8.5%)	30 (8.7%)	>0.9
Extracardiac arteriopathy	198 (5.3%)	175 (5.2%)	23 (6.6%)	0.2
Recent myocardial infarction	140 (3.7%)	119 (3.5%)	21 (6.1%)	0.017
Preoperative cardiac massage	171 (4.6%)	103 (3.0%)	68 (20%)	<0.001
Cardiogenic shock requiring inotropes	643 (17%)	470 (14%)	173 (50%)	<0.001
Penn classification				<0.001
A	2105 (56%)	2001 (59%)	104 (30%)	
B	951 (25%)	890 (26%)	61 (18%)	
BC	419 (11%)	302 (8.9%)	117 (34%)	
C	260 (7.0%)	196 (5.8%)	64 (18%)	
Urgency of the procedure				<0.001
Urgent	515 (14%)	431 (13%)	84 (24%)	
Emergency grade 1	1628 (44%)	1600 (47%)	28 (8.1%)	
Emergency grade 2	1427 (38%)	1259 (37%)	168 (49%)	
Salvage grade 1	98 (2.6%)	66 (1.9%)	32 (9.2%)	
Salvage grade 2	67 (1.8%)	33 (1.0%)	34 (9.8%)	
Salvage procedure	171 (4.6%)	104 (3.1%)	67 (19%)	<0.001
Unconsciousness before sedation	149 (4.0%)	82 (2.4%)	67 (19%)	<0.001
Hemiplegia/hemiparesis	293 (7.8%)	250 (7.4%)	43 (12%)	<0.001
Paraplegia/paraparesis	75 (2.0%)	68 (2.0%)	7 (2.0%)	>0.9
Dysarthria/aphasia	87 (2.3%)	79 (2.3%)	8 (2.3%)	>0.9
Vision loss or disturbances	42 (1.1%)	39 (1.2%)	3 (0.9%)	>0.9
Confusion	375 (10%)	342 (10%)	33 (9.5%)	0.7
Any malperfusion	1371 (37%)	1192 (35%)	179 (52%)	<0.001
Cerebral malperfusion	817 (22%)	682 (20%)	135 (39%)	<0.001
Spinal malperfusion	75 (2.0%)	68 (2.0%)	7 (2.0%)	>0.9
Renal malperfusion	362 (9.7%)	305 (9.0%)	57 (16%)	<0.001
Mesenteric malperfusion	160 (4.3%)	138 (4.1%)	22 (6.4%)	0.045
Peripheral malperfusion	544 (15%)	479 (14%)	65 (19%)	0.019
Tear in the aortic root	625 (17%)	554 (16%)	71 (21%)	0.048
Tear in the ascending aorta	2441 (65%)	2213 (65%)	228 (66%)	0.8
Tear in the aortic arch	616 (16%)	572 (17%)	44 (13%)	0.047
Intramural hematoma	370 (9.9%)	350 (10%)	20 (5.8%)	0.007
Dissection of the aortic root	2390 (64%)	2152 (63%)	238 (69%)	0.051
Dissection of the aortic arch	3135 (84%)	2846 (84%)	289 (84%)	0.8
Dissection of the descending thoracic aorta	1976 (53%)	1785 (53%)	191 (55%)	0.4

eGFR-CKD-EPI = estimated glomerular filtration rate according to the CKD-EPI equation; TAAD = type A aortic dissection. ^1^ Wilcoxon rank sum test was used for continuous variables, whereas Pearson’s Chi-squared test or Fisher’s exact test was used for categorical variables.

**Table 2:** Operative data.

Operative Data	Overall N = 3735	Non-IMV Patients *N* = 3389	IMV Patients *N* = 346	*p*-Value ^1^
Axillary artery cannulation	1598 (43%)	1455 (43%)	143 (41%)	0.6
Femoral and axillary artery cannulation	40 (1.1%)	36 (1.1%)	4 (1.2%)	0.8
Supracoronary replacement	2656 (71%)	2396 (71%)	260 (75%)	0.082
Aortic root replacement	1079 (29%)	993 (29%)	86 (25%)	0.082
Bentall procedure	862 (23%)	781 (23%)	81 (23%)	0.9
David procedure	149 (4.0%)	147 (4.3%)	2 (0.6%)	<0.001
Partial root repair	292 (7.8%)	244 (7.2%)	48 (14%)	<0.001
Coronary artery bypass grafting	338 (9.0%)	296 (8.7%)	42 (12%)	0.035
Mitral valve repair	16 (0.4%)	14 (0.4%)	2 (0.6%)	0.7
Mitral valve replacement	10 (0.3%)	9 (0.3%)	1 (0.3%)	>0.9
Beveled hemiarch repair	1666 (45%)	1520 (45%)	146 (42%)	0.3
Total aortic arch repair	545 (15%)	493 (15%)	52 (15%)	0.8
Frozen elephant trunk procedure	216 (5.8%)	199 (5.9%)	17 (4.9%)	0.5
Myocardial ischema time (min)	110 (80, 152)	109 (80, 151)	117 (83, 158)	0.11
Cardiopulmonary bypass time (min)	204 (162, 260)	204 (162, 260)	207 (155, 268)	>0.9

^1^ Wilcoxon rank sum test was used for continuous variables, whereas Pearson’s Chi-squared test or Fisher’s exact test was used for categorical variables.

**Table 3:** Postoperative outcomes.

Postoperative Outcomes	Overall *N* = 3735	Non-IMV Patients *N* = 3389	IMV Patients *N* = 346	*p*-Value ^1^
Hospital death	647 (17%)	517 (15%)	130 (38%)	<0.001
Ischemic stroke	506 (14%)	461 (14%)	45 (13%)	0.8
Hemorrhagic stroke	69 (1.8%)	49 (1.4%)	20 (5.8%)	<0.001
Global brain ischemia	170 (4.6%)	123 (3.6%)	47 (14%)	<0.001
Paraparesis or paraplegia	194 (5.2%)	171 (5.0%)	23 (6.6%)	0.2
Mesenteric ischemia	143 (3.8%)	121 (3.6%)	22 (6.4%)	0.010
Sepsis	471 (13%)	394 (12%)	77 (22%)	<0.001
Temporary dialysis	408 (11%)	351 (10%)	57 (17%)	<0.001
Permanent dialysis	147 (3.9%)	129 (3.8%)	18 (5.2%)	0.2
Laryngeal nerve palsy	71 (1.9%)	68 (2.0%)	3 (0.9%)	0.14
Reoperation for intrathoracic bleeding	534 (14%)	488 (14%)	46 (13%)	0.6
Tracheostomy	315 (8.4%)	280 (8.3%)	35 (10%)	0.2
Deep sternal wound infection	89 (2.4%)	78 (2.3%)	11 (3.2%)	0.3
V-A-ECMO	109 (2.9%)	81 (2.4%)	28 (8.1%)	<0.001

V-A-ECMO = venoarterial extracorporeal membrane oxygenation. ^1^ Wilcoxon rank sum test was used for continuous variables, whereas Pearson’s Chi-squared test or Fisher’s exact test was used for categorical variables.

**Table 4:** Baseline differences between survivors and in-hospital deaths.

Baseline Characteristics	Overall, *N* = 3735	Survivors *N* = 3088	In-Hospital Deaths *N* = 647	*p*-Value ^1^
Estimated distance to hospital (km)	28 (8, 60)	29 (8, 60)	23 (6, 60)	0.056
Age (years	64 (55, 74)	63 (54, 73)	69 (59, 77)	<0.001
Octogenarians	304 (8.1%)	210 (6.8%)	94 (15%)	<0.001
Female	1140 (31%)	941 (30%)	199 (31%)	0.9
Weight	80 (70, 90)	80 (70, 90)	80 (69, 90)	0.15
Height	174 (165, 180)	174 (165, 180)	172 (165, 180)	0.019
eGFR-CKD-EPI (mL/min/1.73 m^2^)	69 (53, 87)	72 (55, 88)	59 (45, 77)	<0.001
Arterial lactate	1.60 (1.00, 2.80)	1.50 (1.00, 2.50)	2.40 (1.30, 4.50)	<0.001
Genetic syndromes	79 (2.1%)	70 (2.3%)	9 (1.4%)	0.2
Prior cardiac surgery	118 (3.2%)	96 (3.1%)	22 (3.4%)	0.7
Iatrogenic TAAD	102 (2.7%)	72 (2.3%)	30 (4.6%)	0.001
Aspirin	728 (19%)	574 (19%)	154 (24%)	0.002
Oral anticoagulant	248 (6.6%)	191 (6.2%)	57 (8.8%)	0.015
Hypertension	2653 (71%)	2186 (71%)	467 (72%)	0.5
Diabetes	181 (4.8%)	143 (4.6%)	38 (5.9%)	0.2
Prior stroke	145 (3.9%)	113 (3.7%)	32 (4.9%)	0.12
Pulmonary disease	319 (8.5%)	257 (8.3%)	62 (9.6%)	0.3
Extracardiac arteriopathy	198 (5.3%)	146 (4.7%)	52 (8.0%)	<0.001
Recent myocardial infarction	140 (3.7%)	88 (2.8%)	52 (8.0%)	<0.001
Preoperative cardiac massage	171 (4.6%)	89 (2.9%)	82 (13%)	<0.001
Cardiogenic shock requiring inotropes	643 (17%)	458 (15%)	185 (29%)	<0.001
Penn classification				<0.001
A	2105 (56%)	1858 (60%)	247 (38%)	
B	951 (25%)	756 (24%)	195 (30%)	
BC	419 (11%)	287 (9.3%)	132 (20%)	
C	260 (7.0%)	187 (6.1%)	73 (11%)	
Urgency of the procedure				<0.001
Urgent	515 (14%)	463 (15%)	52 (8.0%)	
Emergency grade 1	1628 (44%)	1428 (46%)	200 (31%)	
Emergency grade 2	1427 (38%)	1113 (36%)	314 (49%)	
Salvage grade 1	98 (2.6%)	54 (1.7%)	44 (6.8%)	
Salvage grade 2	67 (1.8%)	30 (1.0%)	37 (5.7%)	
Salvage Procedure	171 (4.6%)	88 (2.8%)	83 (13%)	<0.001
Unconsciousness before sedation	149 (4.0%)	97 (3.1%)	52 (8.0%)	<0.001
Hemiplegia/hemiparesis	293 (7.8%)	224 (7.3%)	69 (11%)	0.003
Paraplegia/paraparesis	75 (2.0%)	55 (1.8%)	20 (3.1%)	0.031
Dysarthria/aphasia	87 (2.3%)	71 (2.3%)	16 (2.5%)	0.8
Vision loss or disturbances	42 (1.1%)	31 (1.0%)	11 (1.7%)	0.13
Confusion	375 (10%)	285 (9.2%)	90 (14%)	<0.001
Any malperfusion	1371 (37%)	1044 (34%)	327 (51%)	<0.001
Cerebral malperfusion	817 (22%)	613 (20%)	204 (32%)	<0.001
Spinal malperfusion	75 (2.0%)	55 (1.8%)	20 (3.1%)	0.031
Renal malperfusion	362 (9.7%)	270 (8.7%)	92 (14%)	<0.001
Mesenteric malperfusion	160 (4.3%)	100 (3.2%)	60 (9.3%)	<0.001
Peripheral malperfusion	544 (15%)	402 (13%)	142 (22%)	<0.001
Tear in the aortic root	625 (17%)	519 (17%)	106 (16%)	0.8
Tear in the ascending aorta	2441 (65%)	2021 (65%)	420 (65%)	0.8
Tear in the aortic arch	616 (16%)	495 (16%)	121 (19%)	0.10
Intramural hematoma	370 (9.9%)	321 (10%)	49 (7.6%)	0.029
Dissection of the aortic root	2390 (64%)	1971 (64%)	419 (65%)	0.7
Dissection of the aortic arch	3135 (84%)	2588 (84%)	547 (85%)	0.6
Dissection of the descending thoracic aorta	1976 (53%)	1604 (52%)	372 (57%)	0.010

eGFR-CKD-EPI = estimated glomerular filtration rate according to the CKD-EPI equation; TAAD = type A aortic dissection. ^1^ Wilcoxon rank sum test was used for continuous variables, and Pearson’s Chi-squared test or Fisher’s exact test was used for categorical variables.

**Table 5:** The urgency of the procedure.

Urgency	Definition
Urgent	Scheduled procedure performed in paucisymptomatic patients, with stable hemodynamic conditions during the index hospitalization, from the next working day after admission.
Emergency grade 1	The procedure is performed in patients with stable conditions and without malperfusion before the beginning of the next working day.
Emergency grade 2	Procedure performed in patients with hemodynamic instability despite the use of inotropes and/or any malperfusion before the beginning of the next working day—no cardiopulmonary resuscitation with chest compression required.
Salvage grade 1	Procedure performed in patients requiring cardiopulmonary resuscitation with external chest compressions and/or open-chest cardiac massage between induction of anesthesia and initiation of cardiopulmonary bypass.
Salvage grade 2	Procedure performed in patients requiring cardiopulmonary resuscitation with external chest compressions en route to the operating theater or before induction of anesthesia.

**Table 6:** Results of regression analysis.

Regression Method	LASSO	MMLR					
Variable	OR	OR	Lower CI	Upper CI	*p*-Value	Estimate	Standard Error
Invasive mechanical ventilation	2.35	2.13	1.60	2.85	<0.0001	0.79	0.15
Coronary artery bypass grafting	1.76	2.52	1.93	3.35	<0.0001	0.65	0.15
Urgency of the procedure	1.63	1.77	1.56	2.00	<0.0001	0.58	0.06
Peripheral malperfusion	1.32	1.40	1.09	1.80	0.0078	0.33	0.13
Year of the procedure	0.61	0.52	0,37	0.74	0.0003	−0.54	0.18
eGFR-CKD-EPI	0.73	0.63	0.57	0.69	<0.0001	−0.48	0.05

CI = confidence interval; eGFR-CKD-EPI = estimated glomerular filtration rate according to the CKD-EPI equation; LASSO = Least Absolute Shrinkage and Selection Operator; MMLR = multilevel multivariable logistic regression; OR = odds ratio.

## Data Availability

The data underlying this article will be shared on reasonable request to the corresponding author.

## Supplementary Materials

The following supporting information can be downloaded at https://www.mdpi.com/article/10.3390/jcdd12070239/s1: Figure S1: Patient inclusion flowchart; Table S1: Variance inflation factor (VIF) for variables included in the multivariable regression model.

## Abbreviations

The following abbreviations are used in this manuscript:

AUC	Area under the curve
CABG	Coronary artery bypass grafting
CI	Confidence interval
CKD	Chronic kidney disease
CKD-EPI	Chronic Kidney Disease Epidemiology Collaboration (formula for eGFR)
CPR	Cardiopulmonary resuscitation
ECMO	Extracorporeal membrane oxygenation
eGFR	Estimated glomerular filtration rate
ERTAAD	European registry of type A aortic dissection
GERAADA	German registry for acute aortic dissection type A
ICU	Intensive care unit
IMV	Invasive mechanical ventilation
IRAD	International Registry of Acute Aortic Dissection
LASSO	Least Absolute Shrinkage and Selection Operator
MMLR	Multilevel multivariable logistic regression
OR	Odds ratio
ROC	Receiver operating characteristic
TAAD	Type A aortic dissection
V-A-ECMO	Venoarterial extracorporeal membrane oxygenation
