# Supplementary material for: Preoperative Mechanical Ventilation Prior to Surgical Repair for Type A Aortic Dissection: Incidence, Risk, and Outcomes
Source: J Cardiovasc Dev Dis. 2025 Jun 23;12(7):239. doi: 10.3390/jcdd12070239 (PMC12295867; doi:10.3390/jcdd12070239)
Supplement: Supplementary file 1 [file jcdd-12-00239-s001.zip › jcdd-3615409-sup.2-table.7.pdf]

Supplementary Table 1. Variance Inflation Factor (VIF) for variables included in the multivariable regression model.

Variable	VIF
age	1.03
eGFR	1.03
arterial_lactate	1.02
cardiogenic_shock	1.02
any_malperfusion	1.01
cerebral_malperfusion	1.02
urgency_procedure	1.00
tear_aortic_root	1.04
aortic_arch_repair	1.04
IMV	1.02

VIF < 5 indicates no significant multicollinearity between variables.
