# Supplementary figures and images for: Preoperative Mechanical Ventilation Prior to Surgical Repair for Type A Aortic Dissection: Incidence, Risk, and Outcomes
Source: J Cardiovasc Dev Dis. 2025 Jun 23;12(7):239. doi: 10.3390/jcdd12070239 (PMC12295867; doi:10.3390/jcdd12070239)

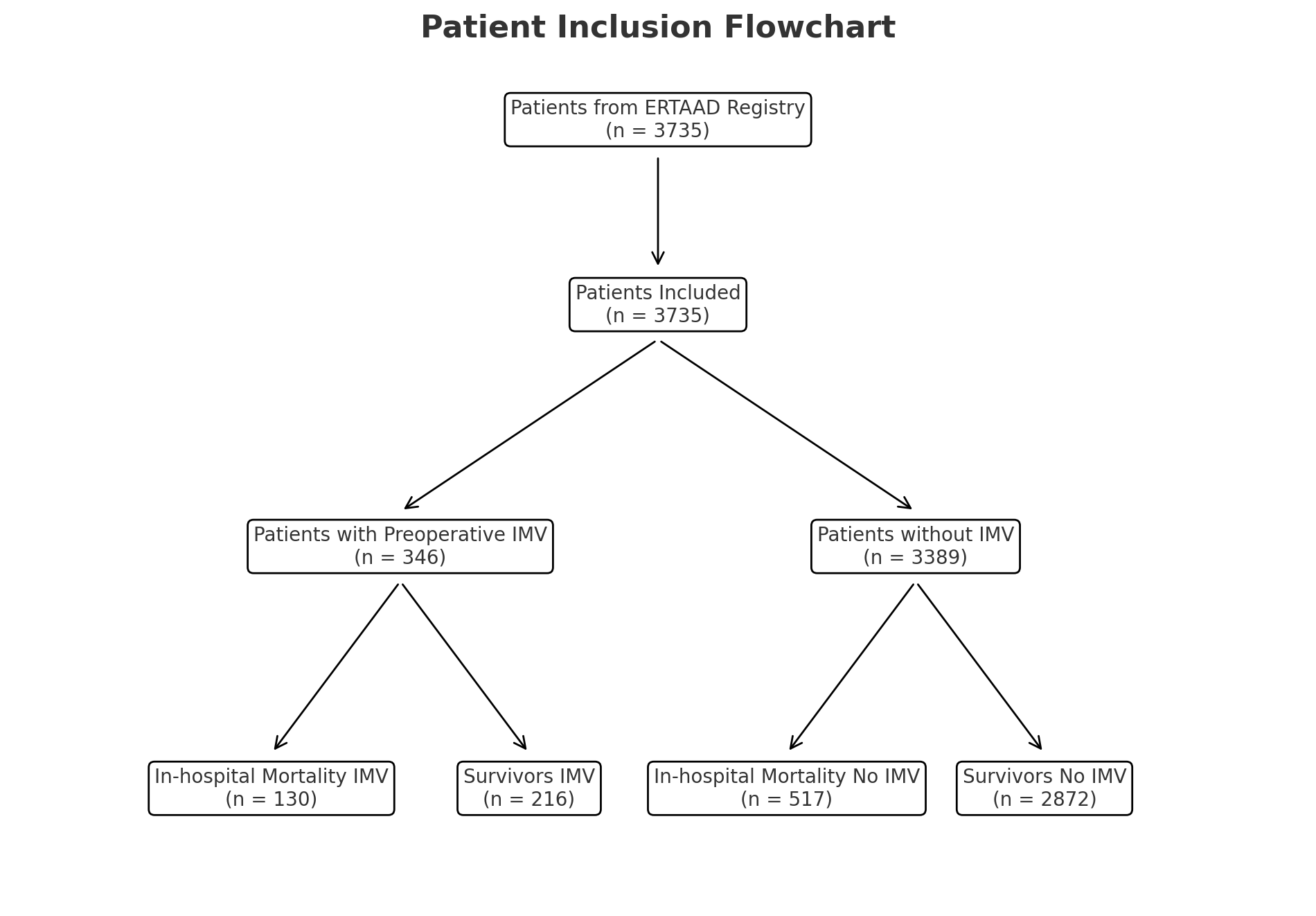

Supplement: Supplementary file 1 [file jcdd-12-00239-s001.zip › jcdd-3615409-sup.1.png]
